# A double SIMEX approach for bivariate random-effects meta-analysis of diagnostic accuracy studies

**DOI:** 10.1186/s12874-016-0284-2

**Published:** 2017-01-11

**Authors:** Annamaria Guolo

**Affiliations:** Department of Statistical Sciences, Via Cesare Battisti 241/243, Padova, Italy

**Keywords:** Bivariate meta-analysis, Diagnostic test, Likelihood inference, Measurement error, SIMEX

## Abstract

**Background:**

Bivariate random-effects models represent a widely accepted and recommended approach for meta-analysis of test accuracy studies. Standard likelihood methods routinely used for inference are prone to several drawbacks. Small sample size can give rise to unreliable inferential conclusions and convergence issues make the approach unappealing. This paper suggests a different methodology to address such difficulties.

**Methods:**

A SIMEX methodology is proposed. The method is a simulation-based technique originally developed as a correction strategy within the measurement error literature. It suits the meta-analysis framework as the diagnostic accuracy measures provided by each study are prone to measurement error. SIMEX can be straightforwardly adapted to cover different measurement error structures and to deal with covariates. The effortless implementation with standard software is an interesting feature of the method.

**Results:**

Extensive simulation studies highlight the improvement provided by SIMEX over likelihood approach in terms of empirical coverage probabilities of confidence intervals under different scenarios, independently of the sample size and the values of the correlation between sensitivity and specificity. A remarkable amelioration is obtained in case of deviations from the normality assumption for the random-effects distribution. From a computational point of view, the application of SIMEX is shown to be neither involved nor subject to the convergence issues affecting likelihood-based alternatives. Application of the method to a diagnostic review of the performance of transesophageal echocardiography for assessing ascending aorta atherosclerosis enables overcoming limitations of the likelihood procedure.

**Conclusions:**

The SIMEX methodology represents an interesting alternative to likelihood-based procedures for inference in meta-analysis of diagnostic accuracy studies. The approach can provide more accurate inferential conclusions, while avoiding convergence failure and numerical instabilities. The application of the method in the R programming language is possible through the code which is made available and illustrated using the real data example.

**Electronic supplementary material:**

The online version of this article (doi:10.1186/s12874-016-0284-2) contains supplementary material, which is available to authorized users.

## Background

Meta-analysis of diagnostic studies is a widely accepted approach for the assessment of the accuracy of a diagnostic test in distinguishing between diseased and nondiseased patients. A diagnostic study is commonly evaluated in terms of sensitivity, i.e., the conditional probability of testing positive in diseased subjects, and specificity, i.e., the conditional probability of testing negative in nondiseased subjects. Alternatively, the information about a diagnostic test is available as a two-by-two table of agreement between the test results and the reference standard test results [1].

The interest in meta-analysis of diagnostic accuracy studies has increased over recent years. Preliminary approaches based on separate univariate meta-analyses for sensitivity and specificity of diagnostic tests, although still diffuse in medical investigations, have been successfully improved by more sophisticated solutions accounting for the correlation between the diagnostic test measures [2–4]. The literature, initially based on least squares regressions [5, 6], now spans hierarchical models [4, 7–9], bivariate copula distributions [10–12], bivariate mixture models [13, 14], nonparametric solutions [15]. In this paper we focus on the bivariate random-effects model [7, 8], as it is currently a well-established and recommended method for meta-analysis of diagnostic accuracy studies. The bivariate random-effects approach has a hierarchical structure accounting for the within-study sampling variability and for the between-study variability arising from differences derived, for example, from patients’ characteristics. Moreover, it considers the presence of measurement error affecting the sample estimation of sensitivity and specificity. These characteristics represent a substantial step ahead with respect to the original approach of Littenberg and Moses [5, 6] to construct a summary receiver operating characteristic (SROC) curve based on the regression of the difference between sensitivity and specificity on their sum, a solution criticised has a source of unreliable inferential conclusions [8]. Likelihood inference has to deal with issues of considerable interest [16–19]: small sample size is known to affect the accuracy of the inferential results; non-convergence of the optimisation algorithms can occur, with non-positive definite variance/covariance matrix or unreliable parameter estimates typically on the boundary of the parameter space; computational issues, such as numerical integration, may represent further complications to deal with.

This paper investigates the applicability of SIMEX (simulation extrapolation) as an alternative way for meta-analysis of diagnostic accuracy studies. SIMEX is a simulation-based technique developed within the measurement error literature [20, 21] that found a wide applicability in many areas of research, given the simplicity of the idea underlying the approach and the straightforward implementation with standard software. The performance of SIMEX for inference on the bivariate random-effects model components as well as on the diagnostic accuracy measures is compared to the likelihood approach through an extensive simulation study covering different scenarios, with varying sample size and between-study correlation. Attention is paid to the robustness of the competing methods against model misspecification, in particular deviations from the typical assumption of joint normal distribution for the random effects [9], as well as to non-convergence problems and numerical instabilities. In addition, SIMEX is applied to the meta-analysis of transesophageal echocardiography recently used in literature [15] to highlight the limitations of the likelihood-based inference.

## Methods

### Bivariate random-effects formulation for meta-analysis

Consider a meta-analysis of *n* diagnostic accuracy studies, each of them providing information as a two-by-two table reporting the number of true positives, true negatives, false positives and false negatives, denoted by *n*_11*i*_,*n*_00*i*_,*n*_10*i*_ and *n*_01*i*_, respectively. Let *n*_1*i*_ be the number of total positives and *n*_0*i*_ the number of total negatives. Consider the sensitivity (*SE*_*i*_) and the specificity (*SP*_*i*_) as diagnostic accuracy measures of study *i,i*=1,…,*n*. Keeping with much of the literature, the accuracy can be expressed using the logit transformation, *η*_*i*_=logit(*SE*_*i*_) and *ξ*_*i*_=logit(1−*SP*_*i*_). Given the two-by-two table information, the estimates of *SE*_*i*_ and *SP*_*i*_ in study *i* are *n*_11*i*_/*n*_1*i*_ and *n*_00*i*_/*n*_0*i*_, respectively. Hereafter, the estimates of *η*_*i*_ and *ξ*_*i*_ will be denoted by $\hat {\eta }_{i}$ and $\hat {\xi }_{i}$, respectively.

#### Models

In this paper, we will focus on the bivariate random-effects model for meta-analysis of diagnostic accuracy studies, following Reitsma et al. [7] and Arends et al. [8], among others. The model has a hierarchical structure, including a within-study level and a between-study level accounting for the correlation between sensitivity and specificity [2–4]. The *between-study model* considers the joint distribution of the random effects *η*_*i*_ and *ξ*_*i*_,

1 $$ \left(\begin{array}{c} \eta_{i} \\ \xi_{i} \end{array} \right) \sim \text{Normal} \left(\left(\begin{array}{c} \overline{\eta} \\ \overline{\xi} \end{array}\right), \left(\begin{array}{cc} \sigma^{2}_{\eta} & \rho \sigma_{\eta} \sigma_{\xi} \\ \rho \sigma_{\eta} \sigma_{\xi} & \sigma^{2}_{\xi} \end{array}\right) \right),  $$

where $\overline {\eta }$ and $\overline {\xi }$ are the means over the studies, $\sigma ^{2}_{\eta }$ and $\sigma ^{2}_{\xi }$ denote the between-study variances and *ρ* is the correlation coefficient. As sensitivity *SE*_*i*_ and specificity *SP*_*i*_ tend to be negatively correlated, then *η*_*i*_ and *ξ*_*i*_ tend to be positively correlated, so that *ρ*>0.

The *within-study variability* is accounted for at the second stage to describe the relationship between $(\hat {\eta }_{i}, \hat {\xi }_{i})^{\top }$ and (*η*_*i*_,*ξ*_*i*_)^⊤^. The literature distinguishes between the approximate and the exact within-study model specification. The approximate model considers $(\hat {\eta }_{i},\hat {\xi }_{i})^{\top }$ following a bivariate normal specification,

2 $$ {}\begin{aligned} &\left(\begin{array}{c} \hat{\eta}_{i} \\ \hat{\xi}_{i} \end{array} \right) \sim \text{Normal} \left(\left(\begin{array}{c} \eta_{i} \\ \xi_{i} \end{array}\right),\right.\\&\qquad \left.\left(\begin{array}{cc} n_{1i}^{-1} + (n_{1i}-n_{11i})^{-1} &0 \\ 0 & n_{0i}^{-1} + (n_{0i}-n_{00i})^{-1} \end{array}\right) \right), \end{aligned}  $$

where the within-study variance/covariance matrix is diagonal with non-zero entries estimated in each study. We refer to (1) and (2) as the *Normal-Normal approach* [16]. Since, marginally,

$${} \begin{aligned} &\left(\begin{array}{c} \hat{\eta}_{i} \\ \hat{\xi}_{i} \end{array} \right) \sim \text{Normal} \left(\left(\begin{array}{c} \overline{\eta} \\ \overline{\xi} \end{array}\right),\right.\\& \left.\left(\begin{array}{cc} \sigma^{2}_{\eta} \,+\, n_{1i}^{-1} \,+\, (n_{1i}\,-\,n_{11i})^{-1} & \rho \sigma_{\eta} \sigma_{\xi} \\ \rho \sigma_{\eta} \sigma_{\xi} & \sigma^{2}_{\xi} \,+\, n_{0i}^{-1}\! +\! (n_{0i}\,-\,n_{00i})^{-1} \end{array} \right) \right), \end{aligned} $$

the likelihood function for $ {\theta }=(\overline {\eta }, \overline {\xi }, \sigma ^{2}_{\eta }, \sigma ^{2}_{\xi }, \rho)^{\top }$ has a closed-form expression and a straightforward implementation using standard softwares. The model has an interesting interpretation in terms of the model suggested by Rutter and Gatsonis [22] within a Bayesian framework, with a different parameterization [23]. From a practical point of view, the implementation of the bivariate Normal-Normal model is, however, more convenient [8].

The exact within-study model specification considers the observed true positives and false positives as realisations of binomial variables,

3 $$ \begin{aligned} &n_{11i} \sim {\text{Binomial}}\left(n_{1i}, (1+e^{-\eta_{i}})^{-1}\right)~ \text{and}\\&\qquad ~ n_{10i} \sim {\text{Binomial}}\left(n_{0i}, (1+e^{-\xi_{i}})^{-1}\right). \end{aligned}  $$

We refer to (1) and (3) as the *Binomial-Normal approach* [16]. The resulting model is a generalised linear model, with no closed-form expression for the associated likelihood function. More computational effort is required with respect to the approximate model, as numerical integration is needed. Convergence problems represent a further drawback of the approach, with the risk of non-positive definite variance/covariance matrix and unreliable estimates of the parameters of the variance/covariance matrix truncated on the boundary of the parameter space [9, 16, 19]. Both the practical issues are more severe as the number of studies decreases. The Normal-Normal approach is prone to some criticism as well, despite its feasible application. Inferential conclusions can be biased as a consequence of small sample size or values of sensitivity and specificity close to 1 [4, 24]. When the sample size is large, instead, there are no substantial differences between the two approaches.

Parameter estimation is typically performed via maximum likelihood or restricted maximum likelihood [8]. The estimates of sensitivity and specificity are obtained by back-transforming the estimates of $\overline {\eta }$ and $\overline {\xi }$, with standard errors derived using the delta method. Alternative measures of test accuracy are the positive likelihood ratio *LR*+=*SE*/(1−*SP*), the negative likelihood ratio *LR*−=(1−*SE*)/*SP* and the diagnostic odds ratio *dOR*={*SE*/(1−*SE*)}×{*SP*/(1−*SP*)}. A description of the diagnostic test can be also provided by the SROC curve, through i) the characterisation of the bivariate normal model via an appropriate line and ii) the transformation of the line to the SROC space. See Arends et al. [8] for alternative specifications for the SROC curves. Discussion about the interpretation of the resulting SROC curve can be found in Hamza et al. [4] and references therein.

#### The measurement error problem

The hierarchical model defined for meta-analysis of diagnostic accuracy studies is an instance of the more general bivariate meta-analysis investigated by van Houwelingen et al. [25], among others. Control rate regression [26, 27], defined as the relationship between the treatment effect and the baseline risk in meta-analysis of clinical trials, perfectly fits the scenario we focus on in this paper. In control rate regression, attention is paid to the risk of inaccurate inferential conclusions due to the presence of measurement error [28, 29] affecting the treatment effect and the baseline risk measures. Different proposals have been suggested to face the measurement error problem [30–32]. Similarly, in meta-analysis of diagnostic accuracy studies the observed $\hat {\eta }_{i}$ and $\hat {\xi }_{i}$ are estimates of the true unknown *η*_*i*_ and *ξ*_*i*_ and thus they are prone to some kind of mismeasure. Not accounting for measurement error can result in misleading inference, the most frequent being a biased estimate of the slope of the regression line used to define the SROC curve, an effect known as *attenuation*. See, for example, the discussion in Arends et al. [8]. The likelihood approach based of the hierarchical model given by (1)–(2) or (1)–(3) properly accounts for measurement errors [8, 26]. The within-study model (2) or (3), in fact, defines a relationship between the observed error-prone $\hat {\eta }_{i}$ and $\hat {\xi }_{i}$ and the unobserved corresponding *η*_*i*_ and *ξ*_*i*_, in this way including the uncertainty related to the measurement process.

Despite the above mentioned analogies, control rate regression and diagnostic accuracy studies differ with respect to the role played by the variables in the regression model. In control rate regression, the baseline risk information is the covariate for the regression model using the treatment effect as response variable. In diagnostic accuracy studies, the roles of *η*_*i*_ and *ξ*_*i*_ in terms of response variable and covariate are not undoubtedly defined, as specificity and sensitivity can act as response or covariate according to the regression model chosen to define a particular SROC curve. Only when a specific regression line used for drawing the SROC curve is defined [8], then the role of response or covariate is clearly stated.

### Double SIMEX approach

SIMEX is a simulation-based technique for measurement error correction [20, 21, 33]. The method, originally developed to deal with classical additive errors affecting continuous variables, can be easily extended to all the scenarios where measurement error structures can be simulated. This requires the measurement error variance to be known or at least accurately approximated. SIMEX consists of a simulation step followed by an extrapolation step. In the first step, a resampling-like strategy simulates *B* datasets of additional errors with increasing variance, each of them used to estimate the parameters of interest. In the second step, the relationship between the estimates and the amount of the added measurement error is determined and used to extrapolate the corrected estimate back to the no measurement error case. The simulation step typically requires the generation of independent random variables, while the estimation can be carried out using standard simple procedures, as the least squares estimation or the method of moments. The extrapolation step is a straightforward procedure. The feasibility of SIMEX application with standard software is the most attractive feature explaining its wide diffusion in many areas of research. Although the approach typically considers one or more mismeasured covariates, SIMEX can easily handle situations with measurement errors on both the response and covariates, see Holcomb [34], who termed the resulting method *double SIMEX*. This case perfectly fits the bivariate meta-analysis problem we focus on in this paper, as $\hat {\eta }_{i}$ and $\hat {\xi }_{i}$ are both affected by measurement error. In this way, SIMEX for diagnostic test accuracy can be thought of as an extension of Guolo [32], who investigated the methodology in control rate regression.

Let $ {W}_{i}=(\hat {\eta }_{i}, \hat {\xi }_{i})^{\top }$ denote the vector of the mismeasured quantities in study *i* and let *X*_*i*_=(*η*_*i*_,*ξ*_*i*_)^⊤^ denote the corresponding error-free vector. In the rest of the paper, we will focus on the approximate within-study model (2) to relate *W*_*i*_ and *X*_*i*_. The implications of using the exact model (3) in SIMEX are illustrated in the Discussion section. The simulation step generates *B* datasets with additional errors starting from *W*_*i*_,

$$ {W}_{b,i}(\lambda)= {W}_{i} + \sqrt{\lambda} {\Gamma}_{i}^{-1/2} {U}_{b,i}, \ b=1, \ldots, B, $$

for fixed *λ*≥0 and *Γ*_*i*_ denoting the variance/covariance matrix of *W*_*i*_. The additional pseudo-errors *U*_*b,i*_ are mutually independent and normally distributed, with zero mean and identity variance/covariance matrix. These properties are not guaranteed in finite sample as the values are computer generated. A possible solution is to simulate the errors from the Gram-Schmid process [34]: the resulting pseudo-errors have the required independence properties and they are normalized to guarantee zero mean and unit variance. Moreover, their use reduces the Monte Carlo variance of the SIMEX estimates, see Section 5.3.4.1 in Carroll et al. [28]. Vector *W*_*b,i*_(*λ*) is a *remeasurement* of *W*_*i*_ with increasing error, such that *W*_*b,i*_(*λ*)=*W*_*i*_ for *λ*=0. The remeasurement is such that *E*(*W*_*b,i*_)=*X*_*i*_ and *Var*(*W*_*b,i*_)=(1+*λ*)*Γ*_*i*_ and the mean squared error *E*[{*W*_*b,i*_(*λ*)−*X*_*i*_}^2^|*X*_*i*_] equals zero for *λ*=−1, the key property of the simulated data. For each simulated *b*-th data set, let $ {\hat {\theta }}_{b}(\lambda)$ be the estimate of *θ* obtained using a standard approach, as if the measurement error was absent. The simulation step concludes with the average of the *B* estimates for fixed $\lambda, {\hat {\theta }}(\lambda)=B^{-1}\sum ^{B}_{b=1} {\hat {\theta }}_{b}(\lambda)$. Usually *λ* assumes values on a grid *Λ*, for example *Λ*={0.0,0.5,1.0,1.5,2.0}. The value of *B* is commonly fixed up to 100 [20, 33]. In the extrapolation step, a relationship between $ {\hat {\theta }}(\lambda)$ and *λ* is established and extrapolated back to the case of no measurement error corresponding to *λ*=−1. The resulting estimate is the SIMEX estimate ${\hat {\theta }}_{SIMEX}$. The most diffuse extrapolation function is the quadratic function, given its numerical stability, see Section 5.3.2 in Carroll et al. [28].

Let $ {s}^{2}_{b}(\lambda)$ be the estimated variance/covariance matrix of ${\hat {\theta }}(\lambda)$ and let $ {s}^{2}(\lambda)=B^{-1}\sum ^{B}_{b=1} {s}^{2}_{b}(\lambda)$. Given the sample variance/covariance matrix $ {s}^{2}_{\Delta }(\lambda)$ of $ {\hat {\theta }}_{b}(\lambda)$, the variance/covariance matrix of the SIMEX estimator is obtained by extrapolating back the relationship between $ {s}^{2}(\lambda) - {s}^{2}_{\Delta }(\lambda)$ and *λ* to the case *λ*=−1, see Stefanski and Cook [21] and Appendix B.4 in Carroll et al. [28].

### Simulation studies

Several simulation studies have been conducted to investigate the performance of SIMEX and compare it to the Normal-Normal and the Binomial-Normal likelihood approaches. Data simulation follows a two-stage procedure. In the first stage, values for *η*_*i*_ and *ξ*_*i*_ are generated according relationship (1) or substituting the normal distribution with a *t* distribution with four degrees of freedom, e.g. [9], or a skew-normal [35] distribution. In the last two cases, the robustness of the results is investigated with respect to departures from the common normality assumption for the random effects, which may sometimes not be appropriate [9]. The chosen skew-normal distribution is such that the mean and the variance correspond to those for the normal case, but the skewness parameter for (*η*_*i*_,*ξ*_*i*_)^⊤^ is increased from (0,0)^⊤^ (the normal case) to (−1.0,0.5)^⊤^ and to (−2,2)^⊤^. In the second stage, the set-up is inspired by the studies in Hamza et al. [16] and Diaz [18]. The within-study numbers of true positives and false positives are simulated using relationship (3). The numbers of diseased subjects *n*_1*i*_ and nondiseased subjects *n*_0*i*_ are generated from a uniform variable on [ 40,200]. The number of studies *n* varies in {10;25} in order to evaluate the methods in case of small to moderate sample sizes. Scenarios with decreasing accuracy are considered, namely, high accuracy $(\overline {\eta },\overline {\xi })^{\top }=(2.94, -2.20)^{\top }$, medium accuracy $(\overline {\eta },\overline {\xi })^{\top }=(1.39, -1.50)^{\top }$ and low accuracy $(\overline {\eta },\overline {\xi })^{\top }=(0.62, -0.85)^{\top }$. Accordingly, (*SE*_*i*_,*SP*_*i*_)^⊤^=(0.95,0.90)^⊤^,(*SE*_*i*_,*SP*_*i*_)^⊤^=(0.80,0.82)^⊤^ and (*SE*_*i*_,*SP*_*i*_)^⊤^=(0.65,0.70)^⊤^,*i*=1,…,*n*. Increasing correlation between *η*_*i*_ and *ξ*_*i*_ is considered, *ρ*∈{0.2;0.6;0.8}. Between-study variances $\sigma ^{2}_{\eta }$ and $\sigma ^{2}_{\xi }$ are fixed equal to 1.2 and 0.5, respectively. One thousand datasets are generated for each combination of sample size, correlation and values of $(\overline {\eta },\overline {\xi })^{\top }$.

The integrals in the Binomial-Normal approach are approximated via a Gauss-Hermite procedure with 100 quadrature points. Inference in the Normal-Normal model is carried out using the restricted maximum likelihood, while inference in the Binomial-Normal model uses the maximum likelihood estimation. Likelihood maximisation, based on the Nelder and Mead algorithm [36], employs the method of moments estimates as starting values. SIMEX considers *B*=100 remeasured data generated using the Gram-Schmid process, *λ* assuming values in *Λ*={0.0,0.5,1.0,1.5,2.0} and the quadratic extrapolation function. Parameter estimation within the simulation step is based on model (2). All the methods are implemented in the R programming language [37].

Methods are compared with respect to bias and estimate of standard error of the estimators of the parameters $\overline {\eta }, \overline {\xi }, \sigma ^{2}_{\eta }, \sigma ^{2}_{\xi }, \rho $ and in terms of the 95% confidence interval for the estimators of the measures of diagnostic accuracy given by the diagnostic odds ratio *dOR*, the positive likelihood ratio *LR*+ and the negative likelihood ratio *LR*−. The performance of the methods in terms of convergence problems is investigated as well. Successful convergence is intended as meeting the criterion convergence (e.g., difference between current and updated estimates less than 0.0001) and positive definite variance/covariance matrix. The results under non-convergence are excluded when summarising the simulation results.

## Results

### Simulation results

Tables 1 and 2 report the simulation results for *n*=10 under the normal, the *t* and the skew-normal specification for (*η*_*i*_,*ξ*_*i*_)^⊤^, by distinguishing the high accuracy scenario and the low accuracy scenario. Results for *n*=25 and results for the medium accuracy scenario are included in the Additional file 1. In all the tables, the non-convergence rate is reported in bold when larger than 5%.

**Table 1:** Simulation results for the high accuracy scenario

Random-effects	*ρ*	$\overline {\eta }$	$\overline {\xi }$	$\sigma ^{2}_{\eta }$	$\sigma ^{2}_{\xi }$	*ρ*	Failure
distribution		bias (s.e.)	bias (s.e.)	bias (s.e.)	bias (s.e.)	bias (s.e.)	rate %
		Normal-Normal					
Normal	0.2	-0.19 (0.33)	0.06 (0.23)	-0.47 (0.46)	-0.13 (0.23)	-0.04 (0.27)	2.5
	0.6	-0.16 (0.33)	0.03 (0.23)	-0.42 (0.47)	-0.12 (0.23)	-0.15 (0.22)	1.8
	0.8	-0.17 (0.34)	0.02 (0.23)	-0.38 (0.48)	-0.10 (0.24)	-0.19 (0.18)	1.8
		Binomial-Normal					
	0.2	0.02 (0.37)	-0.01 (0.24)	-0.10 (0.67)	-0.03 (0.28)	0.02 (0.36)	**6.2**
	0.6	0.02 (0.38)	-0.01 (0.24)	-0.03 (0.71)	-0.02 (0.28)	-0.01 (0.28)	**11.1**
	0.8	0.01 (0.37)	-0.00 (0.24)	-0.03 (0.69)	-0.01 (0.29)	-0.02 (0.20)	**19.5**
		SIMEX					
	0.2	0.08 (0.35)	-0.07 (0.25)	0.05 (0.59)	0.14 (0.30)	-0.03 (0.28)	0.0
	0.6	0.08 (0.35)	-0.07 (0.25)	0.08 (0.59)	0.13 (0.29)	-0.13 (0.23)	0.0
	0.8	0.05 (0.35)	-0.06 (0.25)	0.07 (0.59)	0.14 (0.30)	-0.18 (0.19)	0.0
		Normal-Normal					
*t*	0.2	-0.22 (0.39)	0.09 (0.28)	0.07 (0.72)	0.16 (0.38)	-0.06 (0.26)	1.6
	0.6	-0.23 (0.41)	0.04 (0.28)	0.23 (0.80)	0.18 (0.38)	-0.13 (0.22)	0.7
	0.8	-0.20 (0.41)	0.03 (0.29)	0.21 (0.78)	0.22 (0.41)	-0.17 (0.17)	0.8
		Binomial-Normal					
	0.2	0.02 (0.46)	0.01 (0.30)	0.74 (1.13)	0.34 (0.47)	-0.01 (0.33)	3.6
	0.6	-0.02 (0.48)	-0.01 (0.30)	0.89 (1.20)	0.36 (0.48)	-0.01 (0.24)	**7.9**
	0.8	0.00 (0.47)	0.00 (0.31)	0.82 (1.20)	0.40 (0.53)	-0.03 (0.18)	**14.0**
		SIMEX					
	0.2	0.05 (0.42)	-0.05 (0.31)	0.64 (0.85)	0.52 (0.47)	-0.05 (0.28)	0.0
	0.6	-0.01 (0.43)	-0.07 (0.31)	0.77 (0.90)	0.51 (0.47)	-0.10 (0.22)	0.0
	0.8	0.00 (0.42)	-0.05 (0.31)	0.66 (0.86)	0.52 (0.47)	-0.15 (0.17)	0.0
		Normal-Normal					
Skew-normal	0.2	-0.64 (0.29)	0.17 (0.22)	-0.54 (0.38)	-0.13 (0.22)	0.03 (0.26)	1.4
(low skewness)	0.6	-0.57 (0.30)	0.00 (0.23)	-0.49 (0.40)	-0.13 (0.23)	-0.11 (0.21)	1.8
	0.8	-0.52 (0.31)	-0.10 (0.23)	-0.44 (0.43)	-0.13 (0.22)	-0.17 (0.17)	2.1
		Binomial-Normal					
	0.2	-0.54 (0.32)	0.12 (0.23)	-0.35 (0.48)	-0.05 (0.26)	0.10 (0.33)	**5.4**
	0.6	-0.49 (0.32)	-0.05 (0.24)	-0.28 (0.53)	-0.03 (0.28)	0.03 (0.26)	**10.6**
	0.8	-0.44 (0.34)	-0.16 (0.24)	-0.18 (0.59)	-0.02 (0.29)	0.00 (0.19)	**20.0**
		SIMEX					
	0.2	-0.48 (0.32)	0.06 (0.24)	-0.16 (0.48)	0.11 (0.28)	0.05 (0.27)	0.0
	0.6	-0.42 (0.33)	-0.11 (0.25)	-0.10 (0.51)	0.13 (0.29)	-0.09 (0.22)	0.0
	0.8	-0.38 (0.33)	-0.22 (0.25)	-0.04 (0.54)	0.13 (0.30)	-0.16 (0.18)	0.0
		Normal-Normal					
Skew-normal	0.2	-0.59 (0.30)	0.37 (0.20)	-0.52 (0.40)	-0.19 (0.18)	0.20 (0.23)	1.2
(high skewness)	0.6	-0.44 (0.31)	0.24 (0.22)	-0.44 (0.43)	-0.15 (0.21)	0.01 (0.18)	1.8
	0.8	-0.32 (0.32)	0.15 (0.22)	-0.43 (0.44)	-0.13 (0.21)	-0.11 (0.15)	1.2
		Binomial-Normal					
	0.2	-0.49 (0.32)	0.35 (0.21)	-0.30 (0.51)	-0.13 (0.21)	0.30 (0.29)	**7.6**
	0.6	-0.33 (0.35)	0.22 (0.23)	-0.14 (0.62)	-0.06 (0.26)	0.16 (0.20)	**15.1**
	0.8	-0.19 (0.37)	0.13 (0.24)	-0.08 (0.66)	-0.04 (0.27)	0.05 (0.16)	**25.6**
		SIMEX					
	0.2	-0.43 (0.32)	0.31 (0.21)	-0.13 (0.50)	-0.02 (0.22)	0.22 (0.24)	0.0
	0.6	-0.26 (0.34)	0.17 (0.23)	0.00 (0.55)	0.06 (0.26)	0.03 (0.19)	0.0
	0.8	-0.14 (0.35)	0.08 (0.24)	0.02 (0.57)	0.09 (0.27)	-0.10 (0.16)	0.0

Bias and average of the estimated standard errors (s.e.) for the estimators of $\overline {\eta }, \overline {\xi }, \sigma ^{2}_{\eta }, \sigma ^{2}_{\xi }, \rho $, on the basis of 1, 000 replicates with *n*=10. Failure rates larger than 5% in bold

**Table 2:** Simulation results for the low accuracy scenario

Random-effects	*ρ*	$\overline {\eta }$	$\overline {\xi }$	$\sigma ^{2}_{\eta }$	$\sigma ^{2}_{\xi }$	*ρ*	Failure
distribution		bias (s.e.)	bias (s.e.)	bias (s.e.)	bias (s.e.)	bias (s.e.)	rate %
		Normal-Normal					
Normal	0.2	0.00 (0.32)	0.01 (0.22)	-0.16 (0.51)	-0.07 (0.22)	-0.02 (0.27)	0.5
	0.6	0.00 (0.32)	0.01 (0.21)	-0.19 (0.49)	-0.09 (0.21)	-0.07 (0.20)	0.0
	0.8	0.01 (0.33)	0.01 (0.21)	-0.15 (0.51)	-0.08 (0.21)	-0.08 (0.14)	0.3
		Binomial-Normal					
	0.2	0.02 (0.33)	0.00 (0.22)	-0.09 (0.54)	-0.04 (0.23)	0.00 (0.30)	1.0
	0.6	0.00 (0.33)	0.00 (0.22)	-0.11 (0.53)	-0.05 (0.23)	-0.01 (0.22)	1.4
	0.8	0.01 (0.34)	0.00 (0.22)	-0.05 (0.56)	-0.04 (0.23)	-0.01 (0.14)	4.0
		SIMEX					
	0.2	0.03 (0.34)	-0.01 (0.22)	0.00 (0.54)	0.02 (0.24)	-0.02 (0.28)	0.0
	0.6	0.01 (0.33)	-0.01 (0.22)	-0.02 (0.53)	0.01 (0.23)	-0.06 (0.21)	0.0
	0.8	0.02 (0.34)	-0.01 (0.22)	0.03 (0.55)	0.01 (0.23)	-0.07 (0.14)	0.0
		Normal-Normal					
*t*	0.2	-0.04 (0.40)	0.01 (0.27)	0.44 (0.83)	0.21 (0.37)	-0.02 (0.25)	0.5
	0.6	-0.01 (0.39)	0.03 (0.27)	0.35 (0.78)	0.20 (0.36)	-0.09 (0.20)	0.2
	0.8	-0.01 (0.39)	0.02 (0.27)	0.39 (0.80)	0.21 (0.36)	-0.08 (0.14)	0.2
		Binomial-Normal					
	0.2	-0.01 (0.44)	-0.01 (0.28)	0.80 (1.01)	0.32 (0.42)	0.00 (0.28)	1.3
	0.6	0.01 (0.42)	0.01 (0.28)	0.70 (0.95)	0.32 (0.42)	-0.03 (0.21)	1.2
	0.8	-0.01 (0.43)	0.00 (0.29)	0.81 (1.01)	0.36 (0.43)	-0.01 (0.13)	2.7
		SIMEX					
	0.2	-0.01 (0.44)	-0.03 (0.29)	0.92 (0.96)	0.43 (0.42)	-0.02 (0.26)	0.0
	0.6	0.02 (0.43)	0.00 (0.29)	0.81 (0.91)	0.42 (0.42)	-0.08 (0.20)	0.0
	0.8	0.00 (0.43)	-0.01 (0.29)	0.87 (0.94)	0.47 (0.44)	-0.07 (0.13)	0.0
		Normal-Normal					
Skew-normal	0.2	-0.55 (0.27)	0.13 (0.21)	-0.45 (0.37)	-0.09 (0.21)	0.09 (0.26)	1.0
(low skewness)	0.6	-0.44 (0.29)	-0.02 (0.21)	-0.38 (0.40)	-0.09 (0.21)	-0.05 (0.20)	0.4
	0.8	-0.41 (0.29)	-0.11 (0.21)	-0.34 (0.42)	-0.10 (0.21)	-0.09 (0.14)	0.2
		Binomial-Normal					
	0.2	-0.55 (0.28)	0.12 (0.21)	-0.41 (0.38)	-0.07 (0.22)	0.12 (0.28)	1.8
	0.6	-0.45 (0.30)	-0.03 (0.22)	-0.32 (0.42)	-0.06 (0.23)	0.02 (0.21)	1.8
	0.8	-0.42 (0.31)	-0.12 (0.22)	-0.27 (0.45)	-0.06 (0.23)	-0.01 (0.15)	**5.9**
		SIMEX					
	0.2	-0.55 (0.28)	0.11 (0.22)	-0.35 (0.38)	-0.01 (0.22)	0.09 (0.26)	0.0
	0.6	-0.45 (0.30)	-0.04 (0.22)	-0.26 (0.42)	0.01 (0.23)	-0.04 (0.20)	0.0
	0.8	-0.42 (0.31)	-0.14 (0.22)	-0.21 (0.45)	0.01 (0.23)	-0.08 (0.14)	0.0
		Normal-Normal					
Skew-normal	0.2	-0.51 (0.28)	0.34 (0.19)	-0.41 (0.39)	-0.16 (0.17)	0.26 (0.22)	1.0
(high skewness)	0.6	-0.32 (0.31)	0.23 (0.20)	-0.27 (0.45)	-0.11 (0.20)	0.09 (0.15)	0.1
	0.8	-0.20 (0.31)	0.15 (0.21)	-0.21 (0.48)	-0.09 (0.21)	-0.01 (0.11)	0.2
		Binomial-Normal					
	0.2	-0.51 (0.29)	0.33 (0.19)	-0.36 (0.41)	-0.15 (0.18)	0.31 (0.20)	2.9
	0.6	-0.32 (0.31)	0.23 (0.21)	-0.21 (0.48)	-0.08 (0.21)	0.17 (0.16)	4.1
	0.8	-0.20 (0.33)	0.15 (0.22)	-0.11 (0.53)	-0.05 (0.23)	0.07 (0.10)	**8.5**
		SIMEX					
	0.2	-0.51 (0.29)	0.32 (0.20)	-0.30 (0.40)	-0.10 (0.18)	0.27 (0.22)	0.0
	0.6	-0.32 (0.32)	0.22 (0.21)	-0.13 (0.48)	-0.03 (0.21)	0.11 (0.15)	0.0
	0.8	-0.20 (0.33)	0.14 (0.22)	-0.05 (0.52)	-0.01 (0.22)	0.00 (0.11)	0.0

Bias and average of the estimated standard errors (s.e.) for the estimators of $\overline {\eta }, \overline {\xi }, \sigma ^{2}_{\eta }, \sigma ^{2}_{\xi }, \rho $, on the basis of 1, 000 replicates with *n*=10. Failure rates larger than 5% in bold

Results for the high accuracy scenario (Table 1) show that, for meta-analysis with small sample size and under the random-effects normal specification, the Binomial-Normal approach appears to be preferable in terms of bias of the estimators with respect to alternative solutions, although at the price of a sligthly larger standard error. Such a behaviour, however, deteriorates when moving to *t* and skewed distributions, with the bias tending to increase as the value of the correlation *ρ* becomes smaller. Under a *t* random-effects distribution, the Binomial-Normal approach and SIMEX show an increased bias of the estimators of the variance components $\sigma ^{2}_{\eta }$ and $\sigma ^{2}_{\xi }$, together with an increased standard error. The effects for the Normal-Normal approach are less marked. When considering a skew-normal distribution for (*η*_*i*_,*ξ*_*i*_)^⊤^ with a high value of skewness, SIMEX appears to be the preferable solution in terms of bias, in particular when referring to the estimates of the variance components.

**Fig. 1:**
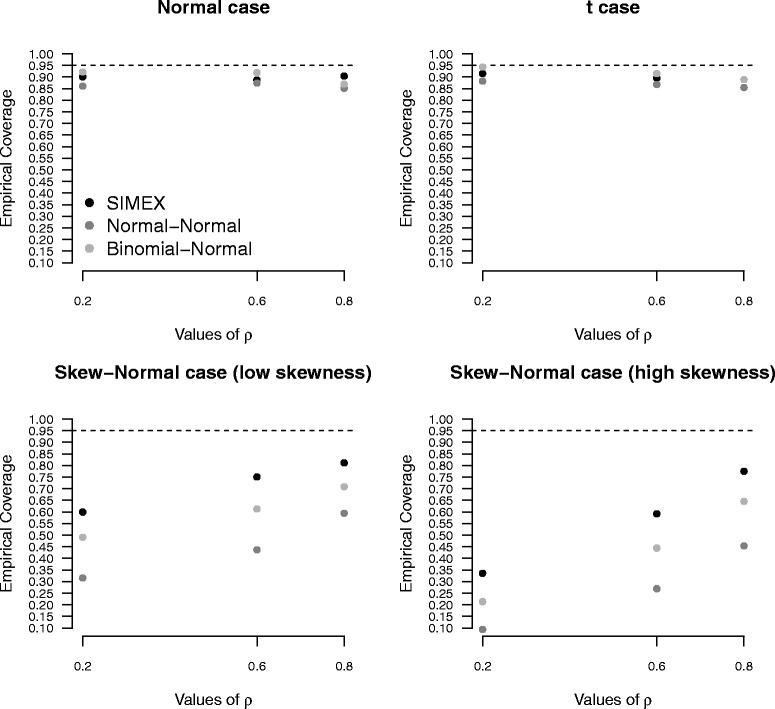
Diagnostic odds ratio results. Empirical coverages of confidence intervals for diagnostic odds ratio under increasing values of *ρ*, on the basis of 1, 000 replicates for the high accuracy scenario, with *n*=10

**Fig. 2:**
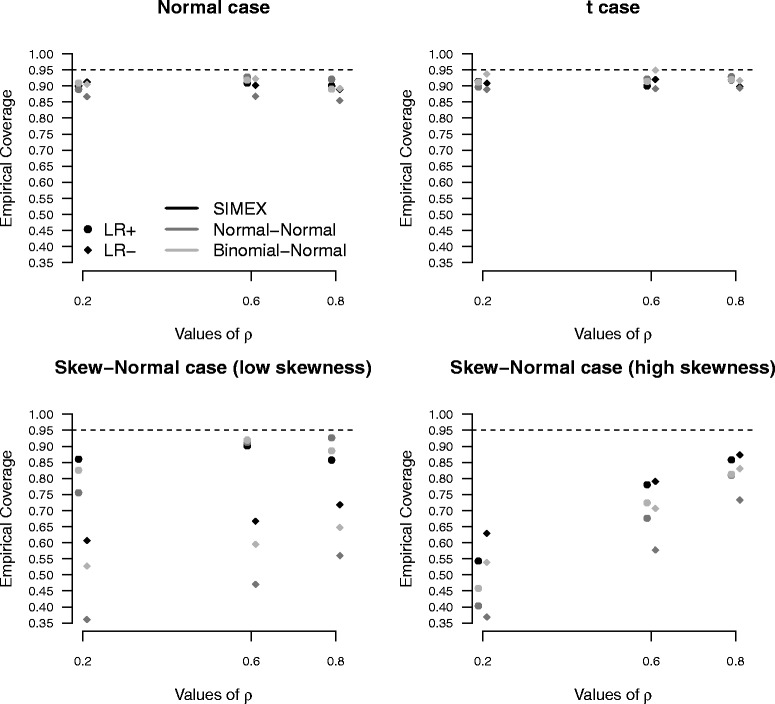
*Positive* and *negative* likelihood ratio results. Empirical coverages of confidence intervals for *positive* and *negative* likelihood ratio under increasing values of *ρ*, on the basis of 1, 000 replicates for the high accuracy scenario, with *n*=10

The impact of misspecification of the random-effects distribution on inferential conclusions becomes more evident when computing the empirical coverage of confidence intervals at 95% nominal level for *dOR* (Fig. 1) and *LR*+ and *LR*− (Fig. 2). For *dOR* (Fig. 1), the Normal-Normal approach and the Binomial-Normal approach provide the less satisfactory results, with empirical coverage probabilities far from the target 95% level, in particular when *ρ* is small and under a skew-normal specification for the random-effects distribution. See, for example, the extreme case *ρ*=0.2 for the high-skewness scenario, with a coverage equal to 10% only for the Normal-Normal approach. Under the normal and *t* scenarios, the performance of the methods is comparable to that of SIMEX, with a slight underestimation of the coverage level for the Normal-Normal method. For skewed scenarios, the advantages of SIMEX over both the likelihood-based solutions are more marked. The empirical coverages of the confidence intervals are substantially closer to the nominal level, especially for large *ρ*. Figure 2 substantiates the results for *LR*+ and *LR*−. Under a normal or *t* specification for (*η*_*i*_,*ξ*_*i*_)^⊤^, methods are still comparable, although using the Normal-Normal likelihood method produces coverages for *LR*− lower than alternatives. For skewed distributions, SIMEX exhibits a more satisfactory behaviour than likelihood solutions in most of the cases when considering *LR*+: for high values of *ρ* and a low skewness, the behaviour is comparable to the other methods. For *LR*−, instead, SIMEX outperforms alternatives, with empirical coverages of confidence intervals closer to the target level as *ρ* increases.

Substantial differences between the competing methods occur in terms of failure rate of the estimation process, see the last column of Table 1. Convergence problems affect the likelihood approach, under the Binomial-Normal formulation in particular, in this way confirming previous findings in the literature [9, 16, 19]. The failure rate is notable when *n*=10, increases with *ρ* and deteriorates under a skew-normal random-effects specification with high values of skewness, thus making the use of the likelihood solution questionable. For the high skewness case and *ρ*=0.8, for example, the Binomial-Normal approach reaches 25.6*%* of failures. More extreme experiments with *ρ*=0.9, not reported here, substantiate the results, with a further growth of non-convergence rate higher than 31%. Conversely from the likelihood approach, the application of SIMEX does not fail in any of the examined situations, irrespectively of the sample size *n*, the correlation *ρ* and the random-effects distribution, with a non-convergence rate constantly equal to zero.

When moving to the low accuracy scenario, results are similar to those observed for the high accuracy case. Consider, for example, results reported in Table 2, where bias and estimated standard error of the estimators are only slightly reduced with respect to Table 1. The most interesting result is the reduction of the failure rate with respect to the high accuracy scenario. With regards to the likelihood analysis, the most substantial reduction of non-convergences is observed for the Binomial-Normal formulation, whose failure rate does not exceed the 5% level under the normal or *t* random-effects formulation and reaches 8.5*%* under the skew-normal distribution. Similarly to the high accuracy context, the failure rate tends to increase with *ρ*. SIMEX maintains a failure rate equal to zero.

Results for the medium accuracy scenario are reported in Additional file 1. Conclusions are coherent wth those from the low and high accuracy scenario. From a computational point of view, non-convergence problems mainly affect the Binomial-Normal approach, with failure rates reaching 22.3% under the skew-normal specification when the sample size is small.

Results for *n*=25 are reported in Additional file 1. Inferential conclusions about the bias of the estimators under all the accuracy scenarios remain globally similar to those for *n*=10, with the advantage of a reduced estimate of the standard error of the estimators. The most interesting result related to the increased sample size is the reduction of the failure rate, under all the examined situations. The Normal-Normal approach is almost convergent in all the simulation settings. The Binomial-Normal approach substantially reduces the number of failures, with just two cases exceeding the 5% threshold, corresponding to the skew-normal case with high skewness and *ρ*=0.8 in the high accuracy and medium accuracy scenarios. SIMEX maintains a failure rate equal to zero.

### Data example

Van Zaane et al. [38] perform a meta-analysis of six diagnostic accuracy studies for the assessment of atherosclerosis in the ascending aorta in patients undergoing cardiac surgery through transesophageal echocardiography in place of the reference-standard method given by epiaortic ultrasound scanning. The available information is reported in Table 3. Data have been recently re-analysed in Zapf et al. [15] through a nonparametric approach.

**Table 3:** Transesophageal echocardiography data [38]

Study	TP	FP	TN	FN
1	3	0	72	25
2	3	0	66	19
3	4	0	56	10
4	0	0	8	6
5	4	1	66	10
6	5	1	49	11

Data includes true positives (TP), false positives (FP), true negatives (TN), false negatives (FN)

The likelihood analysis with the Normal-Normal specification results in estimated variances $\sigma ^{2}_{\eta }$ and $\sigma ^{2}_{\xi }$ almost zero. This affects the evaluation of the variance/covariance matrix for the whole parameter vector. Under the constraint of variances equal to zero, the estimation of the sensitivity and the specificity are concordant with the results obtained in van Zaane et al. [38], using the bivariate random-effects model of Reitsma et al. [7]. The corresponding results are reported in Table 4, together with the associated 95% confidence interval. As shown in Zapf et al. [15], the likelihood analysis using the Binomial-Normal specification does not converge and the likelihood estimation of the correlation *ρ* is on the boundary of the parameter space, equal to 1. These results coincide with those available from our implementation of the likelihood approach. Changing the optimization algorithm and the starting values does not solve the non-convergence problem. The application of SIMEX, conversely, does not pose any convergence issue. The estimates of $\overline {\eta }$ and $\overline {\xi }$ resulting in −1.525 (standard error 0.292) and −4.266 (standard error 0.325), respectively, give rise to the estimates of sensitivity and specificity as reported in Table 4. SIMEX-based confidence intervals for sensitivity and specificity are narrower than those available from the Normal-Normal approach. The results from the nonparametric approach of Zapf et al. [15], close to those from SIMEX, are reported for completeness.

**Table 4:** Data analysis

Method	Sensitivity	Specificity
Normal-Normal model	21 (13, 32)	99 (96, 99)
Binomial-Normal model	–	–
SIMEX approach	17.9 (10.9, 27.8)	98.6 (97.4, 99.3)
Nonparametric model (Zapf et al. [15])	19.0 (11.9, 28.9)	99.4 (97.9, 99.8)

Estimates and 95% confidence intervals (in parentheses) for sensitivity and specificity obtained from different methods for the analysis of transesophageal echocardiography data [38]. Results are multiplied by 100

## Discussion

Results from the simulation studies indicate that SIMEX leads to satisfactory inferential results in a wide range of scenarios. When the normality assumption for the random-effects distribution holds, the method is comparable to the likelihood solutions in terms of bias and estimated standard error of the estimators of the parameters of interest and slightly superior to the Binomial-Normal formulation in terms of empirical coverages of confidence intervals for different diagnostic accuracy measures. When departures from the normality assumptions hold in terms of low or high skewness, then advantages of using SIMEX are much more evident. In particular, empirical coverages of confidence intervals for the diagnostic accuracy measures are closer to the 95% target level than alternatives. The likelihood approach under the Binomial-Normal formulation shows a less satisfactory performance.

A substantial difference between SIMEX and the likelihood approach is in terms of failure rate of convergence. SIMEX has not convergence problems whichever the examined scenario. Conversely, likelihood solutions suffer for convergence difficulties, especially in case of skewed random-effects distribution. The highest levels of failure rate are reached using the Binomial-Normal formulation and they are much more frequent as the sample size is small and the value of the correlation *ρ* increases. Simulation results are in accordance with previous findings in the literature about convergence issues and numerical instabilities of the likelihood approach. Possible solutions evaluated in the simulation studies, including the change of the optimisation algorithm and the change of the starting values [16, 19], only slightly reduce the number of failures. When adopting the SIMEX strategy, several solutions are available in case of convergence failure, although we did not experience such a problem in our study. Possible solutions include the choice of a different estimation method within each *b*-th replication of the simulation step or varying the number of simulated datasets *B*. An additional practical strategy is the visual inspection of the SIMEX components and the direct extrapolation of the points of interest. This strategy is suggested in Section B.4.1 of Carroll et al. [28] when the SIMEX estimated variance/covariance matrix is non-positive definite. Although possible, this is an infrequent case and we did not encounter it in our simulations.

From a strictly practical point of view, the implementation of SIMEX, despite its simulation-based nature, is not involved neither time-consuming and can proceed by taking advantage of simple estimation methods, such as the method of moments. The R [37] code for SIMEX implementation is made available in the Additional file 2 and illustrated in the Additional file 3.

Although the scenarios investigated in the paper do not consider the presence of additional level covariates in model (1), SIMEX can be extended to account for them. In this case, the number of remeasured datasets *B* is recommended to be increased in order to guarantee the results having an acceptable precision, see Section 5.3 in Carroll et al. [28].

In this paper, the model structure used for the simulation step in SIMEX is given by the approximate model (2) in place of the exact model (3). The choice implies that, when necessary, the correction that adds 0.5 to the two-by-two table cells equal to zero is applied. Additional empirical investigations with different correction values show that the 0.5 correction does not impact the results. Such a behaviour is related to the SIMEX procedure, as the correction can affect only the original data, while the remeasured data of the simulation step are not influenced. Simulating the discrete components of the two-by-two table in place of their continuous logit transformations $\hat {\eta }_{i}$ and $\hat {\xi }_{i}$ is theoretically possible. In this case, the measurement error problem affects the classification of the positive/negative results in the two-by-two table, thus being called *misclassification problem*, see Küchenhoff et al. [39]. However, moving from the new generated discrete data to the logit transformations would still be an obligatory step, as the data are necessary for inference in the main model (1). In this case, the 0.5 correction would still apply, not only on the original data but in every case the problem arises within the simulation step. How to circumvent these limitations when simulating from the exact model (3) represents a topic of future research.

## Conclusions

This paper focused on bivariate random-effects models for meta-analysis of diagnostic test accuracy. Attention is paid to the presence of errors affecting the measures of diagnostic accuracy. Standard likelihood-based procedures are shown to be prone to several drawbacks, despite their wide diffusion. The inaccuracy of inferential conclusions for small sample size and in case of misspecification of the random-effects distribution is accompanied by computational issues which seriously affect the applicability of the approach. The SIMEX methodology represents an interesting and promising alternative. Reliable inference properly accounting for the presence of measurement errors is obtained with neither computational effort not numerical instabilities. The satisfactory performance of SIMEX illustrated through extensive simulation experiments is not affected by study characteristics, such as sample size or measurement error correlation. Robustness to departures from normal random-effects distributions is a substantial improvement over standard likelihood solutions. The availability of the R code for a user-friendly implementation of SIMEX is aimed at encouraging its use.

## Additional files

Additional file 1 The file includes a portion of the simulation results comparing the performance of SIMEX and likelihood-based approaches in the medium accuracy scenario and in the low and high accuracy scenarios for sample size *n*=25. (PDF 91 kb)

Additional file 2
R code for applying the SIMEX approach. (R 10.2 kb)

Additional file 3 The file illustrates how to apply SIMEX for data analysis using the R code available in Additional file 2. (PDF 64.4 kb)

## Abbreviations

dOR: Diagnostic odds ratio
FN: False negatives
FP: False positives
LR-: Negative likelihood ratio
LR+: Positive likelihood ratio
s.e.: Standard error
SE: Sensitivity
SIMEX: Simulation extrapolation
SP: Specificity
SROC: Summary operating receiver characteristic
TN: True negatives
TP: True positives
