# Supplementary material for: A double SIMEX approach for bivariate random-effects meta-analysis of diagnostic accuracy studies
Source: BMC Med Res Methodol. 2017 Jan 11;17:6. doi: 10.1186/s12874-016-0284-2 (PMC5225626; doi:10.1186/s12874-016-0284-2)
Supplement: Additional file 1 — The file includes a portion of the simulation results comparing the performance of SIMEX and likelihood-based approaches in the medium accuracy scenario and in the low and high accuracy scenarios for sample size n=25. (PDF 91 kb) [file 12874_2016_284_MOESM1_ESM.pdf]

Additional file 1
for
"A double SIMEX approach for bivariate random-effects meta-analysis of diagnostic accuracy studies"
by Annamaria Guolo

This file reports a portion of the results of the simulation study carried out to evaluate the performance of SIMEX and likelihood-based approaches. Results refer to the medium scenario and to the low and high accuracy scenarios for sample size $n = 25$.

Table 1: Simulation results for the medium accuracy scenario. Bias and average of the estimated standard errors (s.e.) for the estimators of $\overline{\eta}$, $\overline{\xi}$, σ^2_η, σ^2_ξ, ρ, on the basis of 1, 000 replicates with $n = 10$. Failure rates larger than 5% in bold.

Random-effects distribution	ρ	$\overline{\eta}$ bias (s.e.)	$\overline{\xi}$ bias (s.e.)	σ^2_η bias (s.e.)	σ^2_ξ bias (s.e.)	ρ bias (s.e.)	failure rate %
				**Normal-Normal**			
Normal	0.2	-0.05 (0.32)	0.04 (0.21)	-0.20 (0.51)	-0.10 (0.22)	-0.02 (0.26)	0.5
	0.6	-0.02 (0.32)	0.02 (0.21)	-0.22 (0.50)	-0.10 (0.22)	-0.10 (0.21)	0.1
	0.8	-0.01 (0.32)	0.03 (0.21)	-0.26 (0.47)	-0.11 (0.21)	-0.12 (0.15)	0.1
				**Binomial-Normal**			
	0.2	-0.01 (0.34)	0.00 (0.22)	-0.06 (0.57)	-0.05 (0.24)	0.01 (0.31)	2.2
	0.6	0.00 (0.34)	0.00 (0.22)	-0.08 (0.56)	-0.05 (0.24)	-0.01 (0.23)	1.5
	0.8	0.01 (0.33)	0.01 (0.22)	-0.11 (0.55)	-0.05 (0.24)	-0.02 (0.16)	**6.9**
				**SIMEX**			
	0.2	0.01 (0.34)	-0.02 (0.23)	0.06 (0.57)	0.05 (0.25)	-0.02 (0.28)	0.0
	0.6	0.03 (0.34)	-0.03 (0.23)	0.05 (0.57)	0.04 (0.25)	-0.08 (0.22)	0.0
	0.8	0.03 (0.34)	-0.02 (0.23)	0.00 (0.54)	0.04 (0.25)	-0.10 (0.15)	0.0
				**Normal-Normal**			
t	0.2	-0.06 (0.39)	0.05 (0.27)	0.33 (0.79)	0.18 (0.36)	-0.04 (0.25)	0.2
	0.6	-0.06 (0.39)	0.03 (0.27)	0.34 (0.79)	0.18 (0.36)	-0.10 (0.20)	0.2
	0.8	-0.04 (0.39)	0.03 (0.27)	0.35 (0.78)	0.20 (0.37)	-0.10 (0.14)	0.1
				**Binomial-Normal**			
	0.2	0.00 (0.43)	0.01 (0.28)	0.71 (0.99)	0.31 (0.42)	-0.02 (0.29)	1.3
	0.6	-0.01 (0.43)	0.00 (0.29)	0.76 (1.01)	0.33 (0.44)	-0.02 (0.22)	1.8
	0.8	0.00 (0.43)	0.00 (0.29)	0.73 (0.98)	0.35 (0.44)	-0.01 (0.14)	4.7
				**SIMEX**			
	0.2	0.03 (0.43)	-0.02 (0.29)	0.82 (0.92)	0.45 (0.43)	-0.04 (0.27)	0.0
	0.6	0.00 (0.43)	-0.04 (0.30)	0.84 (0.93)	0.46 (0.44)	-0.08 (0.20)	0.0
	0.8	0.01 (0.43)	-0.03 (0.30)	0.81 (0.91)	0.47 (0.44)	-0.08 (0.14)	0.0
				**Normal-Normal**			
Skew-normal (low skewness)	0.2	0.89 (0.29)	-0.55 (0.22)	-0.52 (0.39)	-0.14 (0.22)	0.05 (0.26)	1.5
	0.6	1.00 (0.31)	-0.70 (0.23)	-0.46 (0.42)	-0.12 (0.23)	-0.10 (0.21)	1.6
	0.8	1.05 (0.31)	-0.80 (0.22)	-0.47 (0.41)	-0.15 (0.22)	-0.18 (0.18)	1.9
				**Binomial-Normal**			
	0.2	0.99 (0.31)	-0.61 (0.23)	-0.33 (0.49)	-0.06 (0.26)	0.13 (0.33)	**5.4**
	0.6	1.08 (0.33)	-0.75 (0.24)	-0.24 (0.54)	-0.02 (0.29)	0.03 (0.26)	**9.6**
	0.8	1.14 (0.34)	-0.84 (0.24)	-0.24 (0.60)	-0.04 (0.31)	-0.02 (0.20)	**21.2**
				**SIMEX**			
	0.2	1.05 (0.32)	-0.65 (0.24)	-0.14 (0.49)	0.09 (0.27)	0.06 (0.27)	0.0
	0.6	1.14 (0.33)	-0.81 (0.25)	-0.08 (0.52)	0.15 (0.30)	-0.09 (0.22)	0.0
	0.8	1.20 (0.33)	-0.90 (0.24)	-0.07 (0.52)	0.11 (0.29)	-0.17 (0.18)	0.0
				**Normal-Normal**			
Skew-normal (high skewness)	0.2	0.95 (0.29)	-0.34 (0.20)	-0.52 (0.39)	-0.19 (0.18)	0.21 (0.23)	1.2
	0.6	1.10 (0.31)	-0.48 (0.21)	-0.44 (0.44)	-0.15 (0.20)	0.02 (0.18)	1.3
	0.8	1.23 (0.32)	-0.55 (0.22)	-0.43 (0.44)	-0.13 (0.21)	-0.11 (0.15)	1.2
				**Binomial-Normal**			
	0.2	1.05 (0.32)	-0.36 (0.21)	-0.32 (0.50)	-0.13 (0.21)	0.31 (0.29)	**7.8**
	0.6	1.21 (0.34)	-0.50 (0.22)	-0.15 (0.61)	-0.07 (0.25)	0.17 (0.20)	**16.7**
	0.8	1.35 (0.36)	-0.57 (0.23)	-0.11 (0.63)	-0.04 (0.26)	0.05 (0.16)	**22.3**
				**SIMEX**			
	0.2	1.12 (0.32)	-0.41 (0.21)	-0.13 (0.50)	-0.02 (0.22)	0.23 (0.24)	0.0
	0.6	1.27 (0.34)	-0.55 (0.23)	-0.01 (0.55)	0.04 (0.25)	0.03 (0.18)	0.0
	0.8	1.41 (0.35)	-0.62 (0.24)	0.02 (0.57)	0.09 (0.27)	-0.10 (0.16)	0.0

Table 2: Simulation results for the medium accuracy scenario. Bias and average of the estimated standard errors (s.e.) for the estimators of $\overline{\eta}$, $\overline{\xi}$, σ^2_η, σ^2_ξ, ρ, on the basis of 1, 000 replicates with $n = 25$. Failure rates larger than 5% in bold.

Random-effects distribution	ρ	$\overline{\eta}$ bias (s.e.)	$\overline{\xi}$ bias (s.e.)	σ^2_η bias (s.e.)	σ^2_ξ bias (s.e.)	ρ bias (s.e.)	failure rate %
				**Normal-Normal**			
Normal	0.2	-0.03 (0.21)	0.04 (0.14)	-0.16 (0.34)	-0.07 (0.15)	-0.03 (0.18)	0.7
	0.6	-0.02 (0.21)	0.02 (0.14)	-0.17 (0.33)	-0.06 (0.15)	-0.08 (0.14)	0.0
	0.8	-0.02 (0.21)	0.02 (0.14)	-0.18 (0.32)	-0.07 (0.14)	-0.10 (0.10)	0.0
				**Binomial-Normal**			
	0.2	0.00 (0.22)	0.01 (0.15)	-0.03 (0.37)	-0.02 (0.16)	0.00 (0.21)	1.0
	0.6	0.00 (0.22)	0.00 (0.15)	-0.03 (0.37)	-0.01 (0.16)	0.01 (0.15)	0.0
	0.8	0.00 (0.22)	0.00 (0.15)	-0.04 (0.36)	-0.01 (0.16)	0.01 (0.09)	0.7
				**SIMEX**			
	0.2	0.04 (0.23)	-0.02 (0.15)	0.13 (0.38)	0.10 (0.17)	-0.03 (0.19)	0.0
	0.6	0.03 (0.23)	-0.03 (0.15)	0.13 (0.38)	0.10 (0.17)	-0.06 (0.14)	0.0
	0.8	0.03 (0.23)	-0.03 (0.15)	0.13 (0.38)	0.11 (0.17)	-0.09 (0.10)	0.0
				**Normal-Normal**			
t	0.2	-0.08 (0.26)	0.04 (0.18)	0.38 (0.52)	0.23 (0.24)	-0.02 (0.17)	0.5
	0.6	-0.05 (0.26)	0.03 (0.18)	0.39 (0.52)	0.22 (0.24)	-0.09 (0.13)	0.0
	0.8	-0.04 (0.26)	0.03 (0.18)	0.39 (0.51)	0.20 (0.23)	-0.10 (0.10)	0.0
				**Binomial-Normal**			
	0.2	-0.02 (0.28)	0.00 (0.19)	0.76 (0.63)	0.36 (0.28)	0.02 (0.20)	0.7
	0.6	-0.01 (0.29)	-0.01 (0.19)	0.83 (0.66)	0.37 (0.28)	0.00 (0.14)	0.0
	0.8	-0.01 (0.29)	0.00 (0.19)	0.82 (0.65)	0.35 (0.28)	0.00 (0.09)	0.0
				**SIMEX**			
	0.2	0.00 (0.29)	-0.03 (0.20)	0.99 (0.63)	0.54 (0.30)	0.00 (0.18)	0.0
	0.6	0.02 (0.29)	-0.04 (0.20)	1.04 (0.64)	0.55 (0.30)	-0.06 (0.13)	0.0
	0.8	0.02 (0.29)	-0.03 (0.20)	0.99 (0.63)	0.52 (0.29)	-0.07 (0.09)	0.0
				**Normal-Normal**			
Skew-normal (low skewness)	0.2	0.91 (0.19)	-0.52 (0.14)	-0.50 (0.25)	-0.10 (0.15)	0.05 (0.17)	0.6
	0.6	1.00 (0.20)	-0.69 (0.15)	-0.46 (0.27)	-0.10 (0.15)	-0.11 (0.14)	0.1
	0.8	1.04 (0.20)	-0.80 (0.15)	-0.43 (0.27)	-0.13 (0.14)	-0.17 (0.12)	0.0
				**Binomial-Normal**			
	0.2	1.00 (0.20)	-0.57 (0.15)	-0.33 (0.31)	-0.03 (0.17)	0.13 (0.21)	1.0
	0.6	1.09 (0.21)	-0.74 (0.16)	-0.26 (0.33)	-0.02 (0.18)	0.04 (0.16)	0.5
	0.8	1.11 (0.21)	-0.84 (0.15)	-0.23 (0.34)	-0.05 (0.17)	0.01 (0.12)	4.9
				**SIMEX**			
	0.2	1.07 (0.21)	-0.62 (0.16)	-0.11 (0.31)	0.14 (0.19)	0.06 (0.18)	0.0
	0.6	1.15 (0.22)	-0.80 (0.16)	-0.02 (0.34)	0.19 (0.20)	-0.09 (0.15)	0.0
	0.8	1.18 (0.22)	-0.91 (0.16)	0.01 (0.35)	0.16 (0.19)	-0.17 (0.12)	0.0
				**Normal-Normal**			
Skew-normal (high skewness)	0.2	0.95 (0.19)	-0.34 (0.13)	-0.48 (0.26)	-0.17 (0.12)	0.21 (0.16)	0.1
	0.6	1.12 (0.21)	-0.45 (0.14)	-0.37 (0.30)	-0.12 (0.14)	0.02 (0.12)	0.0
	0.8	1.23 (0.21)	-0.54 (0.15)	-0.35 (0.30)	-0.09 (0.15)	-0.10 (0.10)	0.0
				**Binomial-Normal**			
	0.2	1.05 (0.21)	-0.37 (0.14)	-0.28 (0.32)	-0.12 (0.14)	0.32 (0.19)	0.6
	0.6	1.23 (0.23)	-0.47 (0.15)	-0.12 (0.38)	-0.06 (0.16)	0.18 (0.12)	2.0
	0.8	1.35 (0.23)	-0.56 (0.15)	-0.08 (0.40)	-0.03 (0.17)	0.07 (0.09)	**7.5**
				**SIMEX**			
	0.2	1.11 (0.21)	-0.41 (0.14)	-0.05 (0.33)	0.02 (0.15)	0.23 (0.16)	0.0
	0.6	1.30 (0.23)	-0.51 (0.15)	0.12 (0.38)	0.10 (0.17)	0.03 (0.12)	0.0
	0.8	1.41 (0.23)	-0.61 (0.16)	0.14 (0.39)	0.14 (0.18)	-0.10 (0.10)	0.0

Table 3: Simulation results for the high accuracy scenario. Bias and average of the estimated standard errors (s.e.) for the estimators of $\overline{\eta}$, $\overline{\xi}$, σ^2_η, σ^2_ξ, ρ, on the basis of 1, 000 replicates with $n = 25$. Failure rates larger than 5% in bold.

Random-effects distribution	ρ	$\overline{\eta}$ bias (s.e.)	$\overline{\xi}$ bias (s.e.)	σ^2_η bias (s.e.)	σ^2_ξ bias (s.e.)	ρ bias (s.e.)	failure rate %
				**Normal-Normal**			
Normal	0.2	-0.20 (0.22)	0.06 (0.15)	-0.38 (0.31)	-0.10 (0.15)	-0.05 (0.18)	1.0
	0.6	-0.16 (0.22)	0.04 (0.15)	-0.36 (0.31)	-0.09 (0.15)	-0.14 (0.15)	0.1
	0.8	-0.14 (0.22)	0.02 (0.15)	-0.35 (0.32)	-0.08 (0.15)	-0.17 (0.12)	0.0
				**Binomial-Normal**			
	0.2	0.00 (0.24)	0.00 (0.15)	-0.04 (0.43)	-0.02 (0.18)	0.01 (0.23)	0.8
	0.6	0.01 (0.24)	0.00 (0.16)	-0.02 (0.43)	-0.01 (0.18)	0.01 (0.17)	0.6
	0.8	0.01 (0.24)	0.00 (0.16)	-0.02 (0.43)	-0.01 (0.18)	0.01 (0.12)	3.0
				**SIMEX**			
	0.2	0.06 (0.23)	-0.06 (0.16)	0.18 (0.40)	0.18 (0.20)	-0.04 (0.19)	0.0
	0.6	0.07 (0.23)	-0.06 (0.16)	0.19 (0.40)	0.19 (0.20)	-0.12 (0.15)	0.0
	0.8	0.07 (0.23)	-0.06 (0.16)	0.18 (0.40)	0.19 (0.20)	-0.16 (0.12)	0.0
				**Normal-Normal**			
t	0.2	-0.25 (0.27)	0.09 (0.19)	0.23 (0.50)	0.22 (0.25)	-0.06 (0.18)	0.9
	0.6	-0.19 (0.27)	0.06 (0.18)	0.21 (0.49)	0.19 (0.24)	-0.14 (0.15)	0.0
	0.8	-0.17 (0.27)	0.04 (0.19)	0.25 (0.51)	0.24 (0.26)	-0.16 (0.12)	0.0
				**Binomial-Normal**			
	0.2	-0.02 (0.30)	0.00 (0.20)	0.84 (0.73)	0.39 (0.31)	-0.01 (0.21)	1.5
	0.6	0.00 (0.31)	0.01 (0.20)	0.85 (0.73)	0.36 (0.30)	0.00 (0.16)	0.3
	0.8	0.01 (0.31)	0.00 (0.20)	0.88 (0.74)	0.39 (0.31)	0.00 (0.11)	1.2
				**SIMEX**			
	0.2	-0.01 (0.28)	-0.05 (0.21)	0.86 (0.59)	0.61 (0.32)	-0.05 (0.19)	0.0
	0.6	0.01 (0.28)	-0.04 (0.20)	0.85 (0.59)	0.58 (0.31)	-0.11 (0.15)	0.0
	0.8	0.01 (0.28)	-0.05 (0.21)	0.82 (0.58)	0.59 (0.31)	-0.13 (0.11)	0.0
				**Normal-Normal**			
Skew-normal	0.2	-0.63 (0.19)	0.17 (0.14)	-0.50 (0.25)	-0.11 (0.15)	0.05 (0.17)	0.5
(low skewness)	0.6	-0.55 (0.20)	0.00 (0.15)	-0.45 (0.27)	-0.10 (0.15)	-0.11 (0.14)	0.1
	0.8	-0.51 (0.20)	-0.09 (0.15)	-0.40 (0.28)	-0.12 (0.15)	-0.18 (0.12)	0.0
				**Binomial-Normal**			
	0.2	-0.53 (0.20)	0.12 (0.15)	-0.31 (0.31)	-0.04 (0.17)	0.12 (0.22)	0.7
	0.6	-0.47 (0.21)	-0.05 (0.16)	-0.25 (0.33)	-0.02 (0.18)	0.04 (0.16)	1.2
	0.8	-0.43 (0.22)	-0.14 (0.16)	-0.19 (0.36)	-0.03 (0.18)	0.00 (0.12)	4.5
				**SIMEX**			
	0.2	-0.47 (0.21)	0.07 (0.16)	-0.08 (0.32)	0.15 (0.19)	0.06 (0.18)	0.0
	0.6	-0.40 (0.22)	-0.11 (0.16)	-0.01 (0.34)	0.19 (0.20)	-0.10 (0.15)	0.0
	0.8	-0.37 (0.22)	-0.21 (0.16)	0.05 (0.36)	0.18 (0.20)	-0.17 (0.12)	0.0
				**Normal-Normal**			
Skew-normal	0.2	-0.62 (0.19)	0.35 (0.13)	-0.46 (0.26)	-0.17 (0.12)	0.21 (0.15)	0.0
(high skewness)	0.6	-0.45 (0.21)	0.24 (0.14)	-0.39 (0.29)	-0.12 (0.14)	0.01 (0.12)	0.0
	0.8	-0.33 (0.22)	0.15 (0.15)	-0.33 (0.31)	-0.09 (0.15)	-0.10 (0.10)	0.0
				**Binomial-Normal**			
	0.2	-0.52 (0.21)	0.32 (0.14)	-0.27 (0.33)	-0.12 (0.14)	0.32 (0.18)	0.3
	0.6	-0.34 (0.22)	0.22 (0.15)	-0.14 (0.37)	-0.06 (0.16)	0.16 (0.13)	2.3
	0.8	-0.22 (0.24)	0.13 (0.15)	-0.05 (0.42)	-0.02 (0.18)	0.07 (0.09)	**7.6**
				**SIMEX**			
	0.2	-0.46 (0.21)	0.28 (0.14)	-0.03 (0.34)	0.02 (0.15)	0.23 (0.16)	0.0
	0.6	-0.28 (0.23)	0.17 (0.15)	0.09 (0.37)	0.11 (0.17)	0.02 (0.12)	0.0
	0.8	-0.15 (0.23)	0.08 (0.16)	0.18 (0.40)	0.15 (0.19)	-0.09 (0.10)	0.0

Table 4: Simulation results for the low accuracy scenario. Bias and average of the estimated standard errors (s.e.) for the estimators of $\overline{\eta}$, $\overline{\xi}$, σ^2_η, σ^2_ξ, ρ, on the basis of 1, 000 replicates with $n = 25$. Failure rates larger than 5% in bold.

Random-effects distribution	ρ	$\overline{\eta}$ bias (s.e.)	$\overline{\xi}$ bias (s.e.)	σ^2_η bias (s.e.)	σ^2_ξ bias (s.e.)	ρ bias (s.e.)	failure rate %
				**Normal-Normal**			
Normal	0.2	-0.01 (0.21)	0.01 (0.14)	-0.10 (0.34)	-0.04 (0.15)	-0.03 (0.18)	0.8
	0.6	-0.01 (0.21)	0.00 (0.14)	-0.14 (0.32)	-0.06 (0.14)	-0.06 (0.13)	0.0
	0.8	-0.01 (0.21)	0.00 (0.14)	-0.14 (0.33)	-0.05 (0.14)	-0.08 (0.09)	0.0
				**Binomial-Normal**			
	0.2	0.00 (0.22)	-0.01 (0.15)	-0.03 (0.36)	-0.01 (0.16)	-0.01 (0.20)	1.6
	0.6	-0.01 (0.22)	-0.01 (0.14)	-0.07 (0.35)	-0.03 (0.15)	0.00 (0.14)	0.0
	0.8	-0.01 (0.22)	-0.01 (0.14)	-0.05 (0.35)	-0.02 (0.15)	0.00 (0.09)	0.1
				**SIMEX**			
	0.2	0.01 (0.22)	-0.02 (0.15)	0.08 (0.36)	0.06 (0.16)	-0.03 (0.19)	0.0
	0.6	0.00 (0.22)	-0.02 (0.15)	0.04 (0.35)	0.05 (0.16)	-0.05 (0.14)	0.0
	0.8	0.00 (0.22)	-0.02 (0.15)	0.06 (0.36)	0.06 (0.16)	-0.07 (0.09)	0.0
				**Normal-Normal**			
t	0.2	-0.04 (0.26)	0.02 (0.18)	0.47 (0.53)	0.25 (0.24)	-0.03 (0.17)	0.5
	0.6	-0.01 (0.26)	0.02 (0.18)	0.44 (0.52)	0.25 (0.24)	-0.07 (0.13)	0.0
	0.8	-0.01 (0.26)	0.02 (0.17)	0.39 (0.50)	0.22 (0.23)	-0.07 (0.09)	0.0
				**Binomial-Normal**			
	0.2	-0.02 (0.28)	0.00 (0.19)	0.80 (0.63)	0.36 (0.27)	0.00 (0.19)	1.0
	0.6	0.00 (0.28)	0.01 (0.19)	0.80 (0.63)	0.37 (0.28)	-0.01 (0.14)	0.2
	0.8	-0.01 (0.28)	0.00 (0.19)	0.79 (0.63)	0.36 (0.27)	0.00 (0.08)	0.3
				**SIMEX**			
	0.2	-0.01 (0.29)	-0.01 (0.20)	1.02 (0.63)	0.52 (0.29)	-0.01 (0.18)	0.0
	0.6	0.01 (0.29)	-0.01 (0.20)	1.01 (0.63)	0.52 (0.29)	-0.05 (0.13)	0.0
	0.8	0.00 (0.29)	-0.02 (0.20)	0.99 (0.62)	0.51 (0.29)	-0.05 (0.09)	0.0
				**Normal-Normal**			
Skew-normal (low skewness)	0.2	-0.55 (0.19)	0.13 (0.14)	-0.37 (0.26)	-0.06 (0.14)	0.10 (0.17)	1.1
	0.6	-0.47 0(.19)	-0.03 (0.14)	-0.31 (0.27)	-0.05 (0.14)	-0.03 (0.13)	1.2
	0.8	-0.43 (0.19)	-0.12 (0.14)	-0.29 (0.28)	-0.08 (0.14)	-0.08 (0.09)	0.5
				**Binomial-Normal**			
	0.2	-0.56 (0.19)	0.12 (0.14)	-0.33 (0.27)	-0.04 (0.15)	0.13 (0.19)	2.2
	0.6	-0.48 (0.20)	-0.04 (0.14)	-0.26 (0.29)	-0.02 (0.15)	0.03 (0.14)	1.5
	0.8	-0.44 (0.20)	-0.14 (0.14)	-0.23 (0.30)	-0.04 (0.15)	0.00 (0.09)	0.5
				**SIMEX**			
	0.2	-0.56 (0.19)	0.11 (0.14)	-0.25 (0.27)	0.03 (0.15)	0.10 (0.18)	0.0
	0.6	-0.48 (0.20)	-0.06 (0.15)	-0.19 (0.29)	0.05 (0.16)	-0.02 (0.13)	0.0
	0.8	-0.44 (0.20)	-0.16 (0.14)	-0.14 (0.30)	0.04 (0.15)	-0.07 (0.09)	0.0
				**Normal-Normal**			
Skew-normal (high skewness)	0.2	-0.51 (0.19)	0.34 (0.13)	-0.34 (0.26)	-0.15 (0.11)	0.28 (0.15)	1.0
	0.6	-0.34 (0.20)	0.23 (0.13)	-0.21 (0.30)	-0.09 (0.13)	0.10 (0.10)	0.2
	0.8	-0.21 (0.21)	0.15 (0.14)	-0.17 (0.31)	-0.07 (0.14)	0.00 (0.07)	0.1
				**Binomial-Normal**			
	0.2	-0.51 (0.20)	0.33 (0.13)	-0.29 (0.28)	-0.13 (0.12)	0.33 (0.16)	1.4
	0.6	-0.35 (0.21)	0.22 (0.14)	-0.14 (0.32)	-0.07 (0.14)	0.18 (0.10)	0.6
	0.8	-0.21 (0.22)	0.14 (0.14)	-0.08 (0.34)	-0.03 (0.15)	0.07 (0.06)	0.9
				**SIMEX**			
	0.2	-0.51 (0.20)	0.33 (0.13)	-0.21 (0.28)	-0.08 (0.12)	0.29 (0.15)	0.0
	0.6	-0.34 (0.21)	0.21 (0.14)	-0.06 (0.32)	0.00 (0.14)	0.11 (0.10)	0.0
	0.8	-0.21 (0.22)	0.13 (0.14)	0.02 (0.35)	0.04 (0.15)	0.01 (0.07)	0.0
