# Supplementary material for: A double SIMEX approach for bivariate random-effects meta-analysis of diagnostic accuracy studies
Source: BMC Med Res Methodol. 2017 Jan 11;17:6. doi: 10.1186/s12874-016-0284-2 (PMC5225626; doi:10.1186/s12874-016-0284-2)
Supplement: Additional file 3 — The file illustrates how to apply SIMEX for data analysis using the R code available in Additional file 2. (PDF 64.4 kb) [file 12874_2016_284_MOESM3_ESM.pdf]

Additional file 3
for
"A double SIMEX approach for bivariate random-effects meta-analysis of diagnostic accuracy studies"
by Annamaria Guolo

This file shows how to obtain SIMEX estimates and standard errors for the parameters of the bivariate random-effects model using the software written in the `R` programming language and available in the Additional file 2. The illustration is based on the transesophageal echocardiography data [1] reported in Table 3 of the paper. Functions needed to implement SIMEX can be loaded as follows

```
R> source("Diagnostic_test_SIMEX.R")
```

Data for the analysis:

```
R> TP <- c( 3, 3, 4, 0, 4, 5 ) ## true positives
R> FN <- c( 25, 19, 10, 6, 10, 11 ) ## false negatives
R> TN <- c( 72, 66, 56, 8, 66, 49 ) ## true negatives
R> FP <- c( 0, 0, 0, 0, 1, 1 ) ## false positives
```

SIMEX results are provided by applying function `simex.results`, with arguments

- `TP`: number of true positives
- `FN`: number of false negatives
- `TN`: number of true negatives
- `FP`: number of false positives
- `lambda`: the vector Λ of values for which the simulation step should be done. Default $\Lambda = \{0.0; 0.5; 1.0; 1.5; 2.0\}$
- `B`: number of iterations for each λ. Default `B=100`

- `lambda.first.avoid`: logical, used to include or exclude the first value of Λ, $\lambda = 0$. Default value `FALSE`
- `extr.function`: extrapolation function, to be chosen between `linear` and `quadratic`. Default value `quadratic`

```
## set the seed to guarantee the reproducibility of the results
R> set.seed(0207)
simex.ris.all <- simex.results( TP = TP, FN = FN, TN = TN, FP = FP )
```

The output reports the estimate and the standard error for the component of θ, the associated variance/covariance matrix, the chosen values of λ and the chosen extrapolation function

```
R> simex.ris.all

Simex estimate:
          mu.eta   mu.xi    var.eta  var.xi   rho
Estimate  -1.5250  -4.2663   0.3990   0.3665  -0.3739
Std.Err.   0.2920   0.3255   0.3002   0.3722   0.5958

Simex estimated variance/covariance matrix:
         mu.eta     mu.xi      var.eta    var.xi     rho
mu.eta    0.085291  -0.021686   0.000000   0.000000   0.000000
mu.xi    -0.021686   0.105930   0.001014   0.000000   0.000000
var.eta   0.000000   0.001014   0.090125  -0.012183   0.000000
var.xi    0.000000   0.000000  -0.012183   0.138554  -0.028347
rho       0.000000   0.000000   0.000000  -0.028347   0.354997

Values of lambda:
 0.0 0.5 1.0 1.5 2.0

Extrapolation function: quadratic
```

Additional information reported by the function are the values of the components of θ estimated for each λ.

```
> simex.ris.all$all.theta
       mu.eta     mu.xi sigma2.eta sigma2.xi        rho
0   -1.525346 -4.251527  0.4703852 0.5508821 -0.2702424
```

```
0.5 -1.525485 -4.244906  0.5074810 0.6456262 -0.2286051
1   -1.525542 -4.242164  0.5519832 0.7538321 -0.1918922
1.5 -1.525586 -4.240060  0.5977038 0.8642529 -0.1613759
2   -1.525623 -4.238286  0.6440552 0.9758202 -0.1383087
```

This information can be used for a visual inspection of the SIMEX components and a direct extrapolation of the points of interest, if necessary. For example, the values of $\overline{\eta}$ obtained in the simulation step for the chosen λ are reported in Figure 1, together with the quadratic extrapolation function. The SIMEX estimate corresponding to $\lambda = -1$ is reported with a filled circle. The uncorrected estimate corresponds to $\lambda = 0$.

```
## How to obtain Figure 1
R> plot( c( -1, simex.ris.all$lambda ),
     c( simex.ris.all$theta[,'mu.eta'],
     simex.ris.all$all.theta[,'mu.eta'] ),
     xlab=expression( lambda ), ylab='',
     main=expression( paste( 'Estimate of ', bar( eta ) ) ), las=1 )
## highlight the SIMEX estimate
R> points(-1, simex.ris.all$theta[,'mu.eta'], pch = 19)
## compute the quadratic extrapolation function
R> m <- lm( simex.ris.all$all.theta[,'mu.eta'] ~ simex.ris.all$lambda
         + I( simex.ris.all$lambda^2 ) )
## draw the quadratic extrapolation function
R> curve( coef( m )[1] + coef( m )[2]*x + coef( m )[3]*( x^2 ),
      add=TRUE )
```

Figures

Figure 1: **SIMEX estimate of $\overline{\eta}$.** Estimate of $\overline{\eta}$ for different values of λ and quadratic extrapolation function on the basis of the data in the meta-analysis of Van Zaane et al. [1]. The SIMEX estimate corresponds to $\lambda = -1$.

References

[1] Van Zaane, B., Zuithoff, N.P.A., Reitsma, J.B., Bax, L., Nierich, A.P., Moons, K.G.: Meta-analysis of the diagnostic accuracy of transesophageal echocardiography for assessment of atherosclerosis in the ascending aorta in patients undergoing cardiac surgery. Acta Anaesthesiologica Scandinavia **52**, 1179–1187 (2008)
